# A Brief Note on the Magnetowetting of Magnetic Nanofluids on AAO Surfaces

**DOI:** 10.3390/nano8020118

**Published:** 2018-02-20

**Authors:** Yu-Chin Chien, Huei Chu Weng

**Affiliations:** Department of Mechanical Engineering, College of Engineering, Chung Yuan Christian University, Taoyuan 320-23, Taiwan; vance1829@hotmail.com

**Keywords:** magnetowetting, magnetic fluids, anodic aluminum oxide, contact angle

## Abstract

In magnetowetting, the material properties of liquid, surface morphology of solid, and applied external field are three major factors used to determine the wettability of a liquid droplet on a surface. For wetting measurements, an irregular or uneven surface could result in a significant experimental uncertainty. The periodic array with a hexagonal symmetry structure is an advantage of the anodic aluminum oxide (AAO) structure. This study presents the results of the wetting properties of magnetic nanofluid sessile droplets on surfaces of various AAO pore sizes under an applied external magnetic field. Stable, water-based magnetite nanofluids are prepared by combining the chemical co-precipitation with the sol-gel technique, and AAO surfaces are then generated by anodizing the aluminum sheet in the beginning. The influence of pore size and magnetic field gradient on the magnetowetting of magnetic nanofluids on AAO surfaces is then investigated by an optical test system. Experimental results show that increasing the processing voltage of AAO templates could result in enhanced non-wettability behavior; that is, the increase in AAO pore size could lead to the increase in contact angle. The contact angle could be reduced by the applied magnetic field gradient. In general, the magnetic field has a more significant effect at smaller AAO pore sizes.

## 1. Introduction

Due to the wide potential applications of superhydrophobic and superhydrophilic surfaces, such as self-cleaning [1], anti-icing [2], spray cooling [3], and energy harvesting [4], surface wetting control has been an important issue. In general, the physical and chemical properties of the solid surface are the main reasons for the wettability of a liquid droplet on the surface [5,6,7,8,9]. Different methods have been used to study surface wetting characterization such as measuring the contact angle (CA) of a liquid droplet on surfaces, the work of adhesion of solid-liquid interfaces [10], and the force required to slide the droplet on surfaces [11,12]. The CA measurement technique can be used to investigate how well or how poorly a liquid will spread over a solid surface. If the surface coated with low surface energy molecules (Si, F), a hydrophobic (CA > 90°) performance can be exhibited [13,14,15,16,17]. Otherwise, if the material contains a polar functional group (C=O, COOH, and OH), a hydrophilic (CA < 90°) performance will be enhanced [18]. For physical modification, through the increase of solid surface roughness, resulting in an incremental existence of air in the interface between the liquid droplet and solid surface, a contact angle can be changed from hydrophilic to hydrophobic [19,20,21,22]. However, an uneven surface usually results in a large measurement error. To achieve a regular and homogeneous structure, the method of anodizing an aluminum substrate to form an anodic aluminum oxide (AAO) layer provides is satisfactory. By changing the anodization process parameters, such as electrolyte, temperature, applied voltage, anodizing time, etc., a regular and homogeneous nanostructured AAO surface morphology can be obtained [23,24,25,26]. Ran et al. [27] published a study on the wetting of water droplets on porous alumina surface. Their experiments showed that the wetting phenomenon could change from the Wenzel state to the Cassie state via increasing the pore diameter, which causes a contact angle of 132° at 420 nm pore diameter. They indicated that the CA depends on how much water wets the pores, as well as how much air is trapped and compressed beneath the water. Such a phenomenon of increase in contact angle was also indicated in Kim et al. [28]. 

The material properties of liquid and applied external field are also useful factors that could be used to determine the wettability of a liquid droplet on a surface. Several mechanisms have been proposed to modify the wetting property, including electrowetting, thermocapillary, optowetting, electrowetting-on-dielectric (EWOD), and magnetowetting [29,30,31,32,33]. Among them, the electrowetting-on-dielectric and magnetowetting have become famous research fields because of their fast acting response. Berge [34] published an experimental and theoretical study on the contact angle of water droplets on a solid insulator film under an applied electrical potential, and gave the Young-Lippmann’s equation. Recently, Kim et al. [28] further demonstrated the electrowetting effect of a water droplet on porous metallic nanostructures. They found that the contact angle decreased when the electrical potential increased. Moreover, the electrowettability of porous metallic nanostructures could be further controlled by the pore size. In fact, not only electrowetting but also magnetowetting has a rich phenomenology [35,36]. A magnetic nanofluid is a colloidal suspension of magnetic nanoparticles dispersed in a carrier liquid. For different values of external magnetic field strength, the contact angle and deformation report contrary performances on oil-based magnetic nanfluid and water-based magnetic paint [37]. Rigoni et al. [38] further discussed the interfacial behavior of water-based magnetic nanfluids with various concentrations on a solid surface under different magnetic gradients. Although several research papers present studies on the magnetowetting of magnetic nanofluids under an applied external magnetic field, the effect of surface morphology of solid on the magnetowetting of magnetic nanlofluids has not be investigated.

In this paper, the wetting characteristics of water-based magnetite (Fe_3_O_4_) nanofluid sessile droplets on AAO surfaces are first investigated to analyse the relationship between the contact angle and the pore size. Then, the contact angle as a function of the varying magnetic field gradient is measured using an optical test system.

## 2. Materials and Methods

### 2.1. Preparation of Magnetic Nanofluids

In this study, stable water-based magnetite nanofluids were prepared by combining co-precipitation and sol-gel methods [39,40].

#### 2.1.1. Co-Precipitation Method

Sixteen grams of NaOH were dissolved in 50 mL eionized water and 9.94 g FeCl_2_∙4H_2_O, and 16.22 g FeCl_3_ were dissolved in 100 mL deionized water, so as to obtain 8 M NaOH alkaline solution and iron chloride acid solution (molarity: Fe^2+^/Fe^3+^ = 1/2). After the solutions were mixed and the temperature was cooled down to 25 °C, iron chloride acid solution was titrated with peristaltic pump at 2.823 × 10^−5^ kg/s into alkaline solution with mechanical stirring at 500 rpm. Because the salt was precipitated in reaction process, the magnet was used to adsorb Fe_3_O_4_ magnetic nanoparticles, and the nanoparticles were then washed through decantation process by deionized water. Finally, the magnetic nanoparticles and 12 M NaOH alkaline solution were mixed into 200 mL to complete the unstable water-based magnetite nanofluids.

#### 2.1.2. Sol-Gel Method

An ultrasonic processor was first used to shock the unstable water-based magnetite nanofluids for 10 min, and 5 mL oleic acid was used to cover magnetic particles with peristaltic pump at 2.823 × 10^−5^ kg/s. The mechanical stirring was then set at 500 rpm in process. After titration process was finished, the ultrasonic processor was used to keep shocking for 20 min, then the centrifuge was used to separate large particles from suspended nanoparticles at 5000 rpm for 10 min. Then, the suspensions acted as stable water-based magnetite nanofluids. The volume fraction of particles of the magnetic nanofluids prepared was 2.69%.

### 2.2. Preparation of Anodic Aluminum Oxide Templates

The AAO test templates were made by anodic oxidation process. First, an aluminum sheet (purity: 99.99%; thickness: 0.22 mm) was roughed by wet grinding with 2000 grit. Then, it was cleaned in acetone and ethanol by an ultrasonic cleaner for 5 min and then electropolished in a 4:2:2 volume mixture of H_3_PO_4_, H_2_SO_4_, and deionized water with application of 15 V at 60 °C for 10 min. Next, the first anodic process is during on 0.3 M H_3_PO_4_ solution while applying 20 V, 25 V, 30 V, 35 V, 40 V, 45 V, 50 V, and 55 V at 5 °C for 3 h. Because the random AAO structure is formed in first step, the 6 wt% H_3_PO_4_ solution was used to eliminate oxide layer at 60 °C for 10 min. Then, the second anodic process was conducted under the same condition with first anodization. In each process, the deionized water was used to rinse aluminum sheet. Here, because the AAO test templates were treating in acidic electrolyte. This causes a polar functional group (OH, COOH) to adsorb water molecules using hydrogen bonding and then the wettability on surface were enhanced. Therefore, the AAO test templates were immersed at 25 °C for 3 h using ethanol and baked at 100 °C for 1 h using vacuum oven to break hydrogen bonding. It should be noted that the pore size is proportional to the processing voltage; that is, the processing voltage could be used to characterize the pore size [23,26]. In our experiments, the applied voltage range was between 20 V and 55 V. After Scanning Electron Microscope (SEM; Model Nova NanoSEM 450, FEI, Hillsbora, Oregon, USA) treatments and then image processor (ImageJ) software estimates, the average pore size obtained increased nearly proportionally with increasing voltage from 32.36 nm to 97.62 nm.

### 2.3. Magnetowetting Experimental Setup

**Figure 1:**
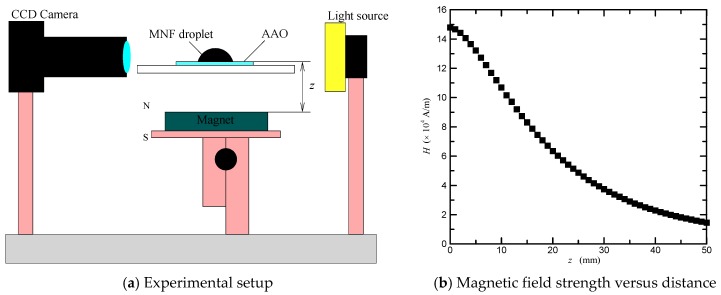
(**a**) Schematic diagram of the experimental setup; (**b**) magnetic field strength away from the magnet’s surface.

To estimate the magnetowetting of magnetic nanofluids, the experimental setup of the optical test system used in this study is shown in Figure 1a. Contact angle of magnetic nanofluid sessile droplet was measured using a contact angle meter (Model 100SL with an accuracy of 0.01 °, Sindatek). All droplets were deposited on AAO surfaces by using a syringe pump. A neodymium magnet was employed to produce an external magnetic field. The magnetic field strength was measured and calculated using a Tesla meter (Model TM-701 with an indication accuracy of ± (5% of measurement value + 0.05) mT, KANETEC, Bensenville, Illinois, USA), and its percentage uncertainty was estimated to be less than 5.25%. The magnetic field strength versus distance is shown in Figure 1b. The magnetic field gradient was obtained by differentiating the strength-distance relation.

## 3. Results and Discussion

**Figure 2:**
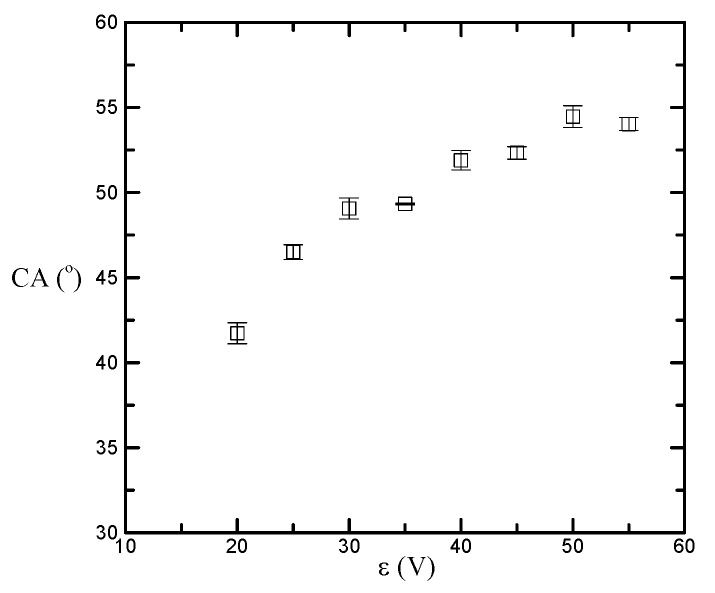
Contact angle versus AAO processing voltage.

In order to understand the effect of surface morphology on the magnetowetting of magnetic nanlofluids on nanostructured AAO surfaces, the measured magnetic nanofluid contact angles with different AAO processing voltages are shown in Figure 2. It can be seen that the contact angle initially increases from 41.73° and then increases slowly to 54.03° when the processing voltage *ε* is changed from 20 V to 55 V at 5 V increments. The percentage increase in contact angle is 29.48%. As mentioned in previous study [27], the larger pore size may lead to an incremental existence of air in the interface between the liquid droplet and solid surface. How much air is trapped and compressed beneath the liquid droplet may, therefore, be the main cause of increased contact angle, and the existence of air in AAO pores appears to have only a limited effect on the wettability of magnetic nanofluids on AAO surfaces.

**Figure 3:**
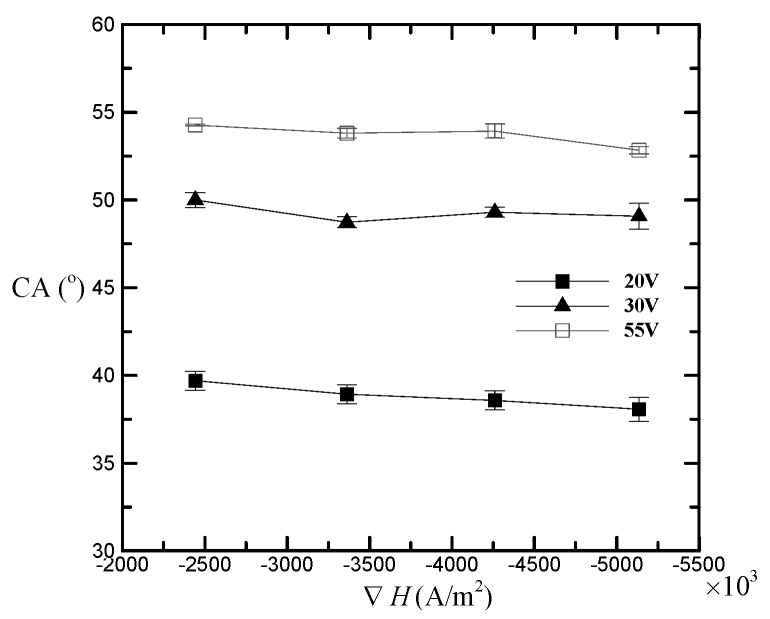
Contact angle changing with applying magnetic field gradient for different AAO processing voltages.

Besides, to investigate the magnetowetting of magnetic nanlofluids on nanostructured AAO surfaces, the influence of magnetic field gradient on the contact angle is shown in Figure 3. From the figure, the AAO surfaces with larger processing voltages show higher contact angles; moreover, regardless of any AAO surfaces, the contact angle generally decreases as the magnetic field gradient increases from −2.4 × 10^6^ A/m^2^ to −5.1 × 10^6^ A/m^2^. The percentage decreases in contact angle for the processing voltages of 20 V, 30 V, and 55 V are 4.11%, 1.86%, and 2.65%, respectively. This means that on a more hydrophobic surface, the applied magnetic field gradient does not necessarily have a more significant effect on the magnetic nanofluid sessile droplet. The reason may be that liquid may be drawn into AAO pores when a magnetic field is applied, which leads to the decrease in contact angle; however, the contact angle also depends on how much air is trapped and compressed beneath the liquid, which leads to the increase in contact angle. Even so, we can still conclude that, in general, the applied magnetic field gradient has a more significant effect on a more hydrophilic surface. 

## 4. Conclusions

Up to now, there was no study evaluating the magnetowetting of magnetic nanofluids on AAO surfaces. The purpose of this study is to investigate experimentally the wetting properties of magnetic nanofluid sessile droplets on surfaces of various AAO pore sizes under an applied external magnetic field. First, stable water-based magnetite nanofluids and AAO templates were prepared by a combined co-precipitation and sol-gel process, and an anodic oxidation process, respectively. Then, to study the effect of pore size on magnetowetting, the contact angle of magnetic nanofluids on the AAO structures was measured using an optical test system magnetically controlled by a neodymium magnet. It was found, from the experiments, that the AAO templates processed by larger voltages show more hydrophobic surfaces. An applied external magnetic field could lead to an increased wetting property. This study may benefit the investigations of the magnetically controllable wettability of droplets in fluidic devices, such as liquid lenses, liquid irises, and biochips.
